# Mycobacterial virulence: impact on immunogenicity and vaccine research

**DOI:** 10.12688/f1000research.20572.1

**Published:** 2019-11-28

**Authors:** Vera M. Kroesen, Jan Madacki, Wafa Frigui, Fadel Sayes, Roland Brosch

**Affiliations:** 1 Unit for Integrated Mycobacterial Pathogenomics, CNRS UMR 3525, Institut Pasteur, Paris, France; 2 Faculty VI, University of Oldenburg, Oldenburg, Germany

**Keywords:** tuberculosis, mycobacteria, host adaption, virulence, pathogen evolution. vaccination

## Abstract

The borderline between virulence and efficacy in live attenuated vaccine strains is often blurred and this is also the case for the Bacillus Calmette–Guérin (BCG), the only currently licensed anti-tuberculosis vaccine used on a large, global scale, which was obtained almost 100 years ago. While BCG is more than 99% identical at the genome level to *Mycobacterium tuberculosis*, the causative pathogen of human tuberculosis, some important differences in virulence factors cause naturally irreversible attenuation and safety of this vaccine in the immunocompetent host. Some of these virulence factors are involved in persistence capacities of the vaccine strains and also represent strong immunogens, responsible for inducing different host signaling pathways, which have to be taken into consideration for the development of revised and new vaccine strains. Here we discuss a number of selected mycobacterial features in relation to their biological functions and potential impact on virulence and vaccine efficacy.

## Introduction

Tuberculosis (TB) remains among the top 10 causes of death worldwide ^1^. In 2017, an estimated 10 million people developed new TB disease, of which 90% were aged 15 or older ^1^. This lacking control of TB in a large number of people emphasizes the complex disease TB represents, involving a highly persistent bacterium, *Mycobacterium tuberculosis*, and the variable abilities of individual human immune systems to cope with this infection. As TB disease may also occur in certain individuals who have been vaccinated during early childhood with the widely used attenuated *Mycobacterium bovis* Bacillus Calmette–Guérin (BCG) vaccine, this situation also points to the shortcomings of BCG, the only licensed vaccine against TB today *.* BCG provides protection against severe, disseminated TB in children, but protection often fades in adolescence and fails to protect against pulmonary TB. The reported efficacy of BCG against adult pulmonary TB varies hugely (0–80%) and seems to depend on many factors, including social conditions, co-infection with helminths, and other variables ^2– 5^. BCG can generally be regarded as safe, although individuals with immune deficiencies can develop disseminated BCG disease ^2^. Thus, despite the millions of childhood TB cases prevented by large-scale BCG vaccination strategies, there is a need for a more effective vaccine that can interrupt the vicious infection cycle of *M. tuberculosis*, which is driven by adolescent and adult patients with pulmonary TB disease. In this review, we will thus focus on selected mycobacterial virulence factors that play a role as potent immunogens as well as potential targets for the attenuation of novel vaccine strains.

## Virulence factors in *Mycobacterium tuberculosis*

An accurate definition of mycobacterial virulence is quite difficult to find, as many factors may influence the survival of *M. tuberculosis* in specific environmental conditions. Apart from essential genes that are needed by *M. tuberculosis* for its survival in commonly used mycobacterial growth media ^6, 7^, its survival in the hostile environment inside host phagocytes requires additional skills from the bacterium. An overview of such *in vivo*-required genes was obtained by using libraries of transposon mutants of *M. tuberculosis* strains in murine infection models, unraveling between 200 and 500 genes essential for the growth of *M. tuberculosis in vivo* ^8, 9^. Among these genes, several were also identified as important virulence determinants of *M. tuberculosis* by comparative analyses of attenuated and virulent tubercle bacilli, including genes encoding proteins involved in secretion systems, persistence mechanisms, or lipid metabolism ^10– 17^, as further discussed below.

### ESX/ESAT-6/type VII secretion systems

ESX secretion systems are specialized bacterial secretion systems needed for the transport of substrates through the complex, thick mycobacterial cell envelope, which is composed of an inner phospholipid bilayer and an outer membrane (also called the mycomembrane) largely composed of mycolic acids, at the inner side covalently attached to arabinogalactan, which is in turn attached to a layer of peptidoglycan embedded in the periplasm, separating the inner and outer membrane ^18, 19^. Variable numbers of these secretion systems are present in mycobacteria and other actinobacteria, such as *Streptomyces* or *Corynebacteria,* and more distantly related ESX-like systems are also found in Gram-positive bacteria, such as *Staphylococcus aureus* ^18, 20^. In mycobacteria, five distinct ESX secretion systems are known to date, ESX-1 to ESX-5, of which at least three, ESX-1, ESX-3, and ESX-5, are needed for full virulence in *M. tuberculosis*. The roles of ESX-2 and ESX-4 are not yet understood ^18^. ESAT-6 (6 kDa early secretory antigenic target/EsxA) as the first identified effector protein has led to the nomenclature of the secretion systems as ESX or ESAT-6 secretion systems, though, of the five ESX secretion systems known to date, only ESX-1 is known to specifically secrete ESAT-6 ^18^. Another commonly used name for ESX systems is type VII secretion (T7S) systems, in analogy to the well-studied secretion systems in Gram-negative bacteria ^21^.

### The ESX-1 secretion system within the region of difference 1 (RD1)

ESX-1, the first-described ESX secretion system, was discovered by comparative genomics approaches between virulent *M. bovis* and/or *M. tuberculosis* and attenuated mycobacterial strains, such as BCG and *Mycobacterium microti* ^10, 22, 23^. Indeed, both BCG and *M. microti* are attenuated members of the *M. tuberculosis* complex (MTBC) that have been widely used for human vaccination ^24, 25^, and, interestingly, both vaccine strains lack overlapping portions of the genomic region of difference 1 (RD1), encoding the ESX-1 secretion system.

The ESX-1 secretion system is a protein complex localized in the mycobacterial plasma membrane. The complex is composed of the ESX conserved components EccB, EccD, and EccE and the ATP-dependent translocase EccC ^26, 27^. The structural organization of the ESX-1 secretion machinery might closely resemble that of the ESX-5 system of *Mycobacterium xenopi*, for which cryoelectron-microscopy-based single particle analysis has revealed a hexameric organization ^28^. Intriguingly, a hexameric structure was also demonstrated for the ESX-3 system of *Mycobacterium smegmatis*, which consists of four protein components: EccB3, EccC3, EccD3, and EccE3 in a 1:1:2:1 stoichiometry ^29^. The main effector protein ESAT-6 is likely secreted by ESX-1 as a heterodimer together with EsxB/CFP-10 (10 kDa culture filtrate protein) in a 1:1 ratio ^30^, although under certain circumstances the two protein partners might also be secreted at other ratios ^16, 27^. As another particularity of the ESX-1 machinery, the secretion of ESAT-6/CFP-10 is dependent on ESX-1-associated proteins EspC and EspA, which in turn seem dependent on the ATPase EccA located in the cytosol and EspD for stabilization ^19, 31– 33^. The functions of EspA and EspC are unknown, but EspC was speculated to form tunnel-like filaments, allowing the secretion of effector proteins through the outer membrane ^34^. Interestingly, the ESX-1-associated EspA, EspC, and EspD are encoded in genomic islands that seem to have been acquired by independent horizontal gene transfer events in several groups of pathogenic mycobacteria at different genomic locations ^35^. EspA and EspC do show some sequence similarity to EspE, EspF, and EspH, which are proteins that are encoded together with the putative chaperone EspG at the 5′ end of the *esx-1* locus. While the deletion of EspF and EspG did not impact the secretion of ESAT-6 and CFP-10, the lack of these proteins attenuated the mutant *M. tuberculosis* strain ^36^. In other experiments, the retained ESAT-6 secretion activity in EspF and EspG deletion mutants was confirmed by using an intraphagocytic release assay ^37^. In addition, *espE* is one of the *esx-1* genes repeatedly identified by large-scale transposon screens as being important for virulence ^8, 9^. There are several other ESX-1-associated proteins encoded by the *esx-1* locus. Sala and co-workers recently reported that EspL is essential for ESX-1-mediated secretion and proposed a model in which EspL associates with EspD to act as a chaperone for EspF, the dimer EspH/EspE, and the dimer EspC/EspA. The latter are needed for the secretion of ESAT-6/CFP-10 ^33^. EspB is another substrate of ESX-1 and has been described to form folds similar to another type of ESX effector protein, namely PE/PPE proteins, containing a similar N-terminal region to PE/PPE. It has been shown that EspB is processed by the protease MycP1 during secretion, and its role in the pathogenicity of *M. tuberculosis* is not yet understood ^19, 38^.

### The PE/PPE protein families and the ESX-5 secretion system

The effector proteins ESAT-6 and CFP-10 secreted by ESX-1 belong to the so-called WXG100 protein family, named after the characteristic WxG hairpin motif ^39^, which is present in the secreted, small Esx proteins of the different ESX systems ^40^. Other important effectors of the ESX secretion apparatus which often also carry a WxG motif belong to the large PE/PPE protein families, which are not specific to ESX-1 ^41^. PE/PPE are polymorphic proteins that share an N-terminal Pro–Glu (PE) or Pro–PE (PPE) signature and similarities in structural folding ^42, 43^. The majority of PE/PPE proteins is secreted by the ESX-5 secretion system, which thus controls the export of a multiplicity of immunogens and potential virulence factors ^14^. The ESX-5 secretion system is the most recently evolved ESX system and is harbored only by slow-growing mycobacteria ^44, 45^. The *esx-5* locus includes five genes encoding secreted PE/PPE proteins, named PPE25 (Rv1787), PE18 (Rv1788), PPE26 (Rv1789), PPE27 (Rv1790), and PE19 (Rv1791), which seem to be the result of gene duplication events of a PE/PPE encoding gene pair, as shown by their high sequence similarity. In addition, many other PE/PPE proteins, which are not directly encoded within the *esx-5* locus, are also exported via the ESX-5 apparatus ^43, 46^. The ESX-5-encoded PE/PPE proteins have been found to be highly immunogenic ^43, 46^ and to represent potential virulence factors, as shown by the attenuation of an *M. tuberculosis* deletion mutant lacking the five *esx-5*-associated *pe/ppe* genes ^14^. In the *M. tuberculosis* H37Rv reference strain, the PPE25, PPE26, and PPE27 proteins belong to the 24-membered PPE-GxxSVPxxW subgroup of PPE proteins ^47^, which share the particular GxxSVPxxW sequence motif. Another prominent member of this subgroup is the PPE18 protein (also known as Rv1196 or Mtb39A), which together with PepA (also known as Rv0125 or Mtb32A) and the adjuvant AS01E constitutes the novel M72/AS01E vaccine candidate, currently in clinical evaluation ^48– 51^. Moreover, another member of the PPE-GxxSVPxxW subgroup is PPE38 (Rv2352c), which was shown to be needed for the export of certain subclasses of PE and PPE proteins in addition to the ESX-5 system ^17^. Indeed, it was found that export of PE_PGRS proteins that are encoded by genes with polymorphic GC-rich sequences, as well as PPE proteins with major polymorphic tandem repeats (PPE-MPTR subgroup), requires the presence of ESX-5 and a functional PPE38 protein. Certain lineages of *M. tuberculosis* strains, such as most of the members of the *M. tuberculosis* lineage 2 (Beijing) strains, lack parts of the genetic region encoding PPE38 and are thus unable to secrete a large number of PE_PGRS and PPE-MPTR proteins ^17^. As phenotypic consequences of such secretion defects, a gain of virulence in certain *M. tuberculosis* strains has been observed in murine infection models ^17^. However, the mechanism by which the secretion of PE_PGRS and PPE_MPTR proteins may interfere with virulence remains unknown. It has also been observed that *M. bovis* and the *M. bovis*-derived BCG vaccine strains lack PPE38 due to the deletion of the RD5 region and therefore do not export the plethora of PE_PGRS and PPE_MPTR proteins ^52^. While genetic introduction of a *ppe38* gene copy into the genome of BCG resulted in recombinant BCG38 showing a re-established secretion phenotype, no significant differences in vaccine efficacy between wild-type BCG and BCG38 have been found in murine infection systems ^52^. However, the finding that the BCG strains currently used in human vaccination do not export certain PE and PPE proteins remains an important discovery for vaccine developers, particularly as certain booster vaccines use PPE proteins as their targets, for which it is uncertain whether they were properly exported by BCG and exposed to the immune system during primary vaccination ^48, 53– 55^. Thus, the elucidation of the function of PE and PPE proteins and their contribution to virulence and immunogenicity remains one main research priority in TB research.

### Lipid surface factors

The outer membrane of mycobacteria is largely composed of mycolic acids and, additionally, it contains several types of non-covalently associated lipids, which, owing to their location, are important players in host–pathogen interaction ^56, 57^. Complex lipids synthesized by *M. tuberculosis* include lipoarabinomannan (LAM), phosphatidylinositol mannosides (PIMs), trehalose monomycolates and dimycolates (TMM, TDM/cord factor), diacyl and polyacyl trehaloses (DAT, PAT), phthiocerol dimycocerosates (PDM, DIM, or PDIM), and sulfolipids (SLs) ^56, 57^.

**Figure 1.**
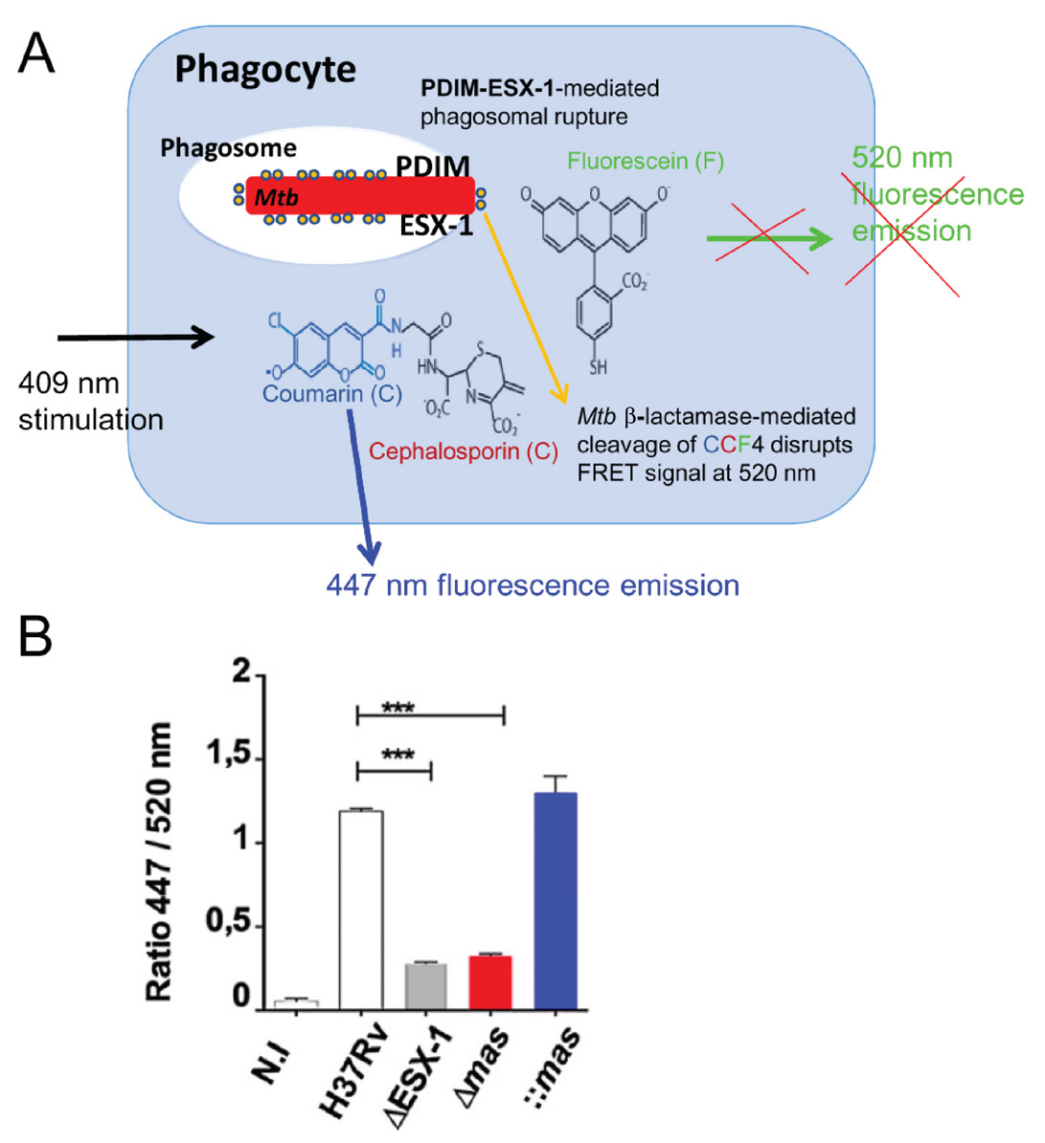
**A**) Cartoon of the CCF4-based fluorescence resonance energy transfer (FRET) assay in phagocytes infected with ESX-1 and phthiocerol dimycocerosate (PDIM) proficient *Mycobacterium tuberculosis* ( *Mtb*) (after ^60^). CCF4-AM (Life Technologies) represents a lipophilic, esterified form of the CCF4 substrate, which allows it to readily enter host cells. Endogenous cytoplasmic esterases rapidly convert CCF4-AM into a negatively charged form, which is trapped in the cytosol. Following excitation at 409 nm, a green fluorescence FRET signal (520 nm) is emitted that is changed into a blue fluorescent signal when phagosomal rupture is induced by *Mtb* via its ESX-1 system in cooperation with the virulence lipid PDIM. By this cytosolic contact, mycobacterial β-lactamase (shown as encircled yellow dots linked to the bacterium) gets in contact with the CCF4 and cleaves it, thereby leading to emission of blue fluorescence at ~450 nm (after ^60^). **B**) Example of results from 15 showing the ratios of fluorescence intensities MFI 447 nm/MFI 520 nm of signals obtained by the above-described FRET assay, implicating *Mtb* ESX-1 and PDIM deletion mutants and complemented strains 6 days after infection of THP-1 cells. Image taken from 15.

PDIMs, for example, have been identified as potent virulence factors of *M. tuberculosis*, as they are required for the induction of phagosomal rupture in the host phagocyte ^13^ in conjunction with the ESX-1 system ^15^ ( Figure 1). Moreover, a recent study suggested that *M. tuberculosis* abundantly releases 1-tuberculosinyladenosine (1-TdAb), a secreted lipid that acts as an antacid and lysosomotropic agent ^58^. Additionally, comparative genomics between pathogenic *M. tuberculosis* and tubercle bacilli with smooth colony morphology, named *M. canettii*, have uncovered a recombination event in the *pks5* genomic region that has apparently led to the loss of lipooligosaccharides (LOS) in the outer membrane of *M. tuberculosis* during the evolution of the MTBC ^59^. This loss of LOS production, which is associated with the appearance of the rough colony morphology in the MTBC members, is thus thought to have contributed to increased fitness and virulence in MTBC members compared to early branching *M. canettii* strains ^59, 61^.

### The antigen 85 complex

The antigen 85 (Ag85) protein complex consists of a set of secreted fibronectin-binding proteins (Fbps), which are essential for maintaining the integrity of the mycobacterial cell envelope. Namely, Ag85 complex is involved in catalyzing the attachment of mycolic acids to arabinogalactan and the biosynthesis of cord factor. While there are four fbp genes ( *fbpA–D*) present in the genome of *M. tuberculosis* H37Rv ^62^, only three of them, Ag85A–C (FbpA, Rv3804c; FbpB, Rv1886c; and FbpC, Rv0129c), have shown mycoloyltransferase activity in complementation experiments ^63^. The same three Ag85A–C proteins also display an Arg–Arg (RR) secretion signal for the twin arginine translocation (TAT) pathway, known to transport folded proteins through the bacterial inner membrane ^64^. Moreover, the Ag85 complex is involved in the invasion of host cells, binding to fibronectin (Fn), tropoelastin, and elastin, essential parts of the extracellular matrix, and, in persistence, preventing maturation of the phagosome ^65– 67^.

**Figure 2.**
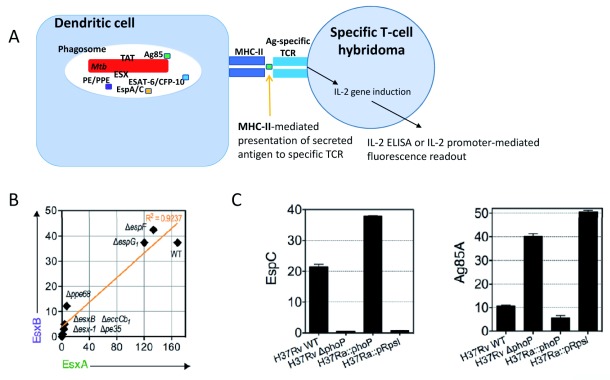
**A**) Cartoon of an intraphagocytic antigen release assay, which uses major histocompatibility complex (MHC)-II-mediated signaling to antigen-specific T-cell hybridoma for the evaluation of secretion of selected proteins as shown for the examples depicted in panels B and C (after ^37^). **B**) Correlation of EsxB (also known as CFP-10) versus EsxA (also known as ESAT-6) intraphagocyte secretion profiles across a panel of various *Mycobacterium tuberculosis* ( *Mtb*) wild-type and mutant strains, reused with permission from 37. **C**) Example of ESX-1 or twin arginine translocation (TAT) signals obtained by intraphagocytic antigen release assays for *Mtb* H37Rv and *Mtb* H37Ra, wild-type, and mutant strains (image taken from 37), which differ in the gene encoding the PhoP two-component virulence regulator ^77^ and hence in the secretion profiles of substrates that are secreted by the TAT pathway. The TAT system is regulated by PhoP via a small RNA (Mcr7), as described in 76. Ag, antigen; CFP-10, 10 kDa Culture Filtrate Protein; ELISA, enzyme-linked immunosorbent assay; ESAT-6, 6 kDa early secretory antigenic target; TCR, T-cell receptor.

The strong immunogenicity of the Ag85 proteins has made them privileged targets for vaccine development. Ag85A, for example, was often used as an expression construct for booster vaccines based on attenuated viral vectors such as modified vaccinia virus Ankara (MVA) or adenovirus ^68, 69^, although an Ag85A-based booster strategy did not enhance protection in a clinical trial over the protection conferred by BCG ^70^. Ag85B was also used as a potential vaccine enhancer. Indeed, a recombinant BCG strain that over-expressed Ag85B induced increased protection in preclinical animal models ^71^ and was also tested in initial clinical trials ^72^. Most interestingly, an increased secretion of Ag85B was also observed in the MTBVAC vaccine candidate, which is currently in clinical evaluation ^73, 74^. This attenuated *M. tuberculosis* strain, in which the *phoP* and the *fadD26* genes were deleted in order to attenuate the strain ^75^, shows enhanced secretion of Ag85B, which is due to an unexpected PhoP-regulatory loop involving a small ncRNA that modulates the secretion of TAT-secreted proteins, such as Ag85 proteins ^76^. In agreement, intraphagocytic release of Ag85B was found to be strongly enhanced in an *M. tuberculosis* PhoP deletion mutant in a dedicated study ^37^ ( Figure 2). This example underlines once more that active export and secretion of mycobacterial antigens outside the bacterial cell are crucial for the appropriate recognition of these antigens by the host immune system, a phenomenon that was also observed for ESX antigens ^37, 77, 78^. This observation also explains in part why heat-killed mycobacterial vaccines have much less immunisation- and protection-capacity than do live attenuated strains, which secrete a wide panel of protective antigens ^79, 80^.

### Virulence factors expressed during latency/latency antigens

*M. tuberculosis* is able to switch to a dormant state/latency during stress conditions. In order to design a vaccine also providing protection against latent *M. tuberculosis* infection (LTBI), antigens expressed during latency need to be considered or antigen presentation during latency needs to be stimulated ^4, 81^. Zhang *et al*. recently reported that EspI, a protein encoded by *esx-1*, is indeed not essential for ESX-1 secretion during active infection but is responsible for down-regulating ESX-1 secretion during low-energy states in the bacilli. This gives important implications for the role of EspI in latency. An EspI knock-out mutant in long-term infection might be rendered incapable of tuning down the secretion of highly immunogenic ESAT-6 under stress conditions, which might result in a stronger immune response against dormant *M. tuberculosis* ^82^.

## Host–pathogen interaction, virulence, and immunogenicity

Most successful vaccines act against infectious agents which can be defeated by humoral immune responses, while it has been proven difficult to design successful vaccines that rely on cellular immune responses, such as in TB ^4^. It is undisputed that in TB the CD4 ^+^ Th1 response is the major factor conferring protection ^1, 83– 85^. Only fairly recently has evidence emerged that other immune cell subsets such as CD8 ^+^ T cells, NK cells, Th17 cells, or B cells may provide an important contribution to protection ^4, 86– 90^. Other factors are well known to play a destructive role, such as Th2 cells and T-regulatory cells/secreted IL-10. Moreover, active TB disease appears to be associated with strong recruitment of neutrophils and type I interferon (IFN) signaling, though the role of type I IFNs in TB remains disputed ^4, 91– 94^. The innate immune response has a crucial role in protection, especially in early infection control ^95, 96^.

ESAT-6 causes phagosomal rupture in the infected host cells, permitting cytosolic contact of the mycobacteria or their secreted products ^18, 60, 97^. The results of this process are varied and may also have consequences for infection control.

**Figure 3.**
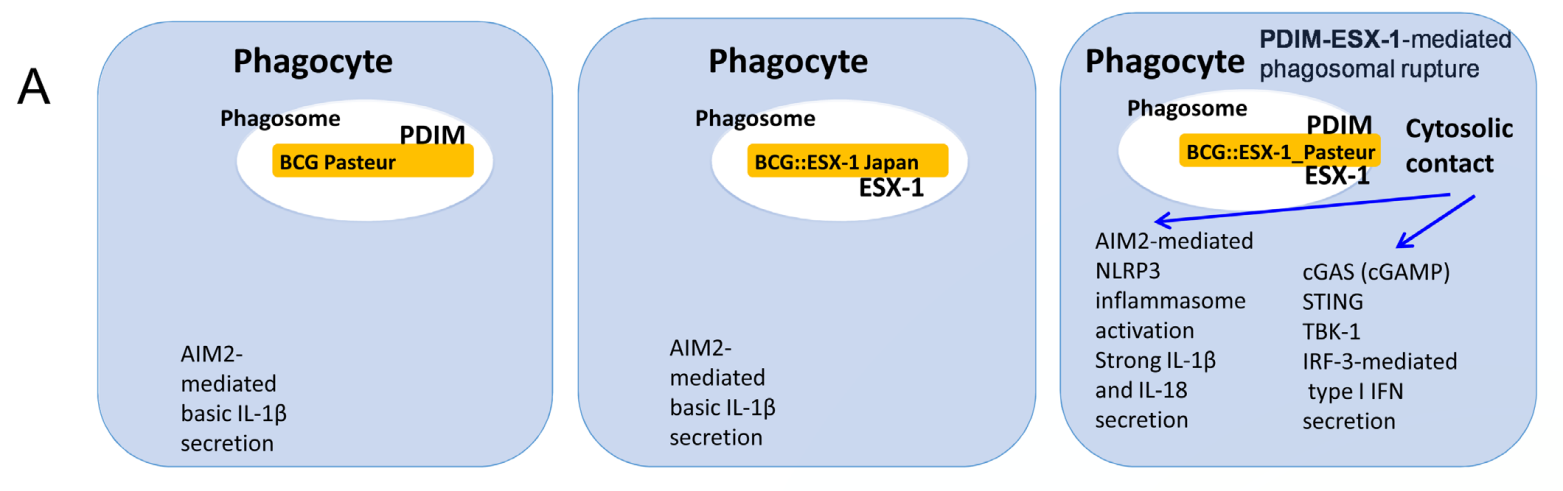
Cartoon showing the impact of the presence of a functional ESX-1 machinery and the outer membrane lipid phthiocerol dimycocerosate (PDIM) on phagosomal rupture for wild-type and recombinant Bacille Calmette–Guérin (BCG) strains, taking into account the results of several recent studies ^15, 86, 113^. The enhanced activation of cytosolic DNA-mediated innate immune signaling of selected recombinant BCG strains is shown. Cytosolic contact via the cooperation between the ESX-1 secretion system and PDIM is achieved only when both components ESX-1 and PDIM are combined and results in the enhancement of induced CD4 ^+^ and CD8 ^+^ T responses, which is correlated with improved protection against a challenge in various preclinical mouse models of infection ^86, 113, 114^. AIM2, absent in melanoma 2; cGAS, cyclic GMP-AMP synthase; IFN, interferon; IL, interleukin; IRF, interferon regulatory factor; STING, stimulator of interferon genes; TBK-1, TANK-binding kinase 1.

Mycobacterial dsDNA that is likely released because of phagosomal rupture into the cytosol is sensed by absent in melanoma 2 (AIM2), which induces the NLRP3 inflammasome to activate caspase-1, which in turn induces the cleavage of pro-IL-1β and pro-IL-18 to active, pro-inflammatory IL-1β and IL-18 ^18, 98^. Moreover, the link between cytosolic access of mycobacteria and type I IFN signaling has clearly been established. dsDNA in the cytosol activates cyclic GMP-AMP synthase (cGAS), leading to the signaling of the second messenger cGAMP, which activates the STING pathway (stimulator of IFN genes). The latter is located at the endoplasmic reticulum and triggers Tank-binding kinase 1 (TBK-1), which activates IFN regulatory factor 3 (IRF3), leading to the production of IFN-β ( Figure 3). Importantly, cGAMP also activates neighboring cells for the production of type I IFN, augmenting the production of IL-10 and IL-1Ra in these bystander host cells, leading to increased host cell necrosis and tissue damage ^18, 94, 99– 101^. On the other hand, Banks *et al*. recently reported a protective bactericidal effect of IFN-β, activating nitric oxide (NO) production, which has been shown to act in a bactericidal and anti-inflammatory manner, leading to killing of the bacilli and preventing tissue damage ^94^. Banks *et al*. suggest a scenario in which *M. tuberculosis* inhibits possibly beneficial autocrine IFN-β signaling while allowing possibly detrimental paracrine IFN-β signaling, while non-pathogenic mycobacteria such as *M. smegmatis* cannot inhibit autocrine IFN-β signaling, which is why the beneficial properties of autocrine type I IFN signaling predominate ^94, 102^ *.* It is probable that the role for type I IFN will not be black and white in TB but ambivalent, for example, depending on the timing of signaling during infection (early, late) or the mode of signaling (autocrine, paracrine).

It has been shown that virulent *M. tuberculosis* H37Rv induces necrosis of infected host cells, leading to damage to the mitochondrial inner and outer membrane, and it has been proposed that this could be mediated by ESAT-6, which also leads to the induction of type I IFN by sensing of mitochondrial DNA, while non-virulent H37Ra causes damage only to the outer membrane of host mitochondria, not leading to necrosis ^103^. Necrosis is an uncontrolled process of cell death and a destructive process in mycobacterial infection. Meanwhile, apoptosis represents a highly controlled process of cell death. Parts of the apoptotic cell as well as mycobacteria disassemble, safely packaged in apoptotic bodies, and are eventually taken up via efferocytosis by phagocytes. This process prevents tissue damage and spread of the bacilli and may enhance the activation of naïve professional antigen-presenting cells (APCs), especially dendritic cells (DCs), taking up mycobacterial components. However, this may also lead to infection. The “fate” of the phagocyte is largely dependent on the cocktail of cytokines present, Th1 cytokines being correlates of protection. A Th1-dominated cytokine cocktail produces classically activated macrophages (CAM, M1) and the production of NO, promoting killing of the bacilli and augmenting the Th1 response, while a Th2-dominated cocktail produces alternatively activated macrophages (AAM, M2) that express arginase-1, are impaired in bacterial killing, and augment Th2 responses ^104^. Moreover, Th1 cytokines stimulate autophagy while Th2 cytokines inhibit autophagy ^103, 105– 107^. Autophagy has been described as an essential process in the control and clearance of mycobacterial infection, as mice lacking the essential autophagy factor Atg5 are highly susceptible to infection ^108, 109^. However, this process may also be entirely due to excess neutrophilic recruitment and resulting tissue damage in ΔAtg5 mice, not to a lack in autophagy, as another study with mice deficient in several players of the autophagy pathway in macrophages, other than Atg5, did not show any higher susceptibility to *M. tuberculosis* ^18, 110^. In the latter study, autophagy in macrophages appeared to be insignificant for the outcome of infection with *M. tuberculosis.* However, this finding might have been influenced by the fact that macrophages are not the most important effectors of autophagy compared to DCs, as DCs are needed for the priming of naïve Th1 cells, which eventually produce IFN-γ to activate phagocytes for the clearance of the bacilli ^110^. Evidence has pointed towards a scenario in which ESAT-6 inhibits autophagy at the step of phagosome–lysosome fusion, resulting in reduced secretion of IL-12 in DCs needed for the induction of Th1, which could be restored by rapamycin therapy ^111, 112^. However, cytosolic access of mycobacteria may also stimulate autophagy via marking of mycobacterial compounds in the cytosol with ubiquitin for selective autophagy or via the AIM2/inflammasome pathway, as seen with the vaccine candidate VPM-1002 ^115^. Moreover, cytosolic contact of mycobacteria via the AIM2/inflammasome/IL-18 pathway has been reported as an essential mechanism for the production of IFN-γ by *M. tuberculosis*-independent memory CD8 ^+^ T cells and NK cells, thus emerging as an important, immediate, and unexpected source of Th1 cytokines ^86^.

A recombinant BCG expressing the ESX-1 from *M. tuberculosis* provided improved protection over BCG but, as a downside, was also more virulent in murine models and therefore not an easily usable vaccine candidate ^10, 114^. Following this observation, the idea to recombine BCG with the ESX-1 of less-virulent mycobacteria such as *M. marinum* was proposed, which resulted in the construction of a recombinant BCG expressing ESX-1 ^*Mmar*^, which confers improved protection compared to BCG in different murine models. This candidate vaccine induced higher levels of CD4 ^+^/CD8 ^+^ T-cell responses, likely through the establishment of cytosolic contact via the heterogeneous ESX-1 ^*Mmar*^ system, which induces innate and adaptive signaling events that are not induced by standard BCG strains ^113^ ( Figure 3), while remaining at a similar safety level as parental BCG.

The picture of how exactly ESAT-6 influences intracellular signaling remains an unsolved puzzle. In the context of *M. tuberculosis* infection, ESAT-6 represents a harmful virulence factor, but, in the context of vaccination with recombinant BCG, ESAT-6 acts as a potent immunogen and initiator of important, additional signaling pathways ^114, 116^.

For the ESX-5-associated PE/PPE proteins, strong immunogenicity has been demonstrated in murine models, inducing CD4 ^+^ and CD8 ^+^ T-cell responses. Importantly, this could also be induced via cross-reactivity by non-ESX-5-associated PE/PPE proteins in a knock-out mutant not expressing the ESX-5-associated PE/PPE, thanks to the redundancy of PE/PPE proteins in the mycobacterial genome and the conservation of the essential ESX-5 secretion system needed for the secretion of a multiplicity of PE/PPE proteins ^43, 46^. The exact function of PE/PPE proteins remains largely unknown, but they have been proposed to be involved in mycobacterial capsule/cell-wall integrity, nutrient uptake, antigenic variation, immunodominance, mycobacterial growth in macrophages, and inhibition of phagosome maturation through the induction of phagosomal rupture ^14, 43, 117, 118^. Thus, certain PE/PPE proteins might have an impact on intracellular signaling that is similar to that of ESAT-6/CFP-10 discussed above, while other features such as nutrient uptake may not be such an important factor for immunogenicity but an interesting target to be considered for the attenuation of possible vaccine candidates.

Lipid surface factors such as pathogen-associated molecular patterns play a crucial role during the early phase of infection, aiding the establishment and persistence of infection. They interact with various types of pathogen recognition receptors (PRRs) on phagocytes, which can lead to the priming of phagocytes, the production of chemokines/cytokines for the recruitment of other players of the immune system, and the production of antimicrobial products or can reversely hamper activation of the phagocyte and mediate the silent entry of the bacilli ^119, 120^. One of the most thoroughly studied lipid surface factors is cord factor/TDM, which was first described in the 1950s ^121^. TDM stimulates PRRs, inducing the production of cytokines and NO, influencing granuloma formation, and playing an important role as an adjuvant; however, it may also be involved in the delay of phagosome maturation ^57, 122, 123^. Cord factor is abundantly present in all mycobacteria, although subtle differences in mycolate structure seem to play a role in the virulence and pathogenicity of varying mycobacterial species ^57^. Other lipid surface factors such as DAT and PAT are associated only with mycobacteria belonging to the MTBC and SL is present only in *M. tuberculosis* strains ^124^. Synthesis of DAT, PAT, and SL needs specific polyketide synthase systems (PKSs). DAT and PAT are associated with the delay of phagosome maturation, and SL has been described as a TLR-2 antagonist, which makes PKSs suitable targets for possible attenuation and drugs ^57, 125, 126^. Other surface lipids have been lost in the MTBC, such as the above-mentioned LOS, because of a recombination event in the *pks5* locus ^59, 127^. Interestingly, the lineage 4 strains of the Euro-American lineage of *M. tuberculosis* strains harbor a frameshift mutation in the *pks15/1* gene, which is not present in lineage 2 Beijing strains and in turn leads to the synthesis of phenolic glycolipids (PGLs) in these latter strains ^57, 128, 129^. This phenomenon has been postulated to be linked with increased virulence of Beijing strains ^130^, although other lineage-specific factors also seem to interfere ^131^. Lipid surface factors thus play a dual role in mycobacteria as immune response boosters, as virulence factors, or even as attenuation factors and could simultaneously be employed as vaccine adjuvants, as drug targets, or for attenuation ^119^. Moreover, lipid surface factors can directly activate the adaptive immune responses via recognition by CD1; the exact role of this alternative activation of T-cells will need to be further elucidated ^132, 133^.

## Perspectives on vaccine research

There is some skepticism over whether it will be possible to develop a truly effective vaccine against TB, but there is also some evidence that gives hope for a positive outcome. First of all, it is the existence of BCG, which does provide excellent protection against miliary and meningeal TB in children. Second, it is the fact that previous *M. tuberculosis* infection or controlled LTBI does seem to provide some protection against the development of active TB disease after reinfection, as 90–95% of people infected with *M. tuberculosis* exhibit a protective immune response and as a result do not develop active TB in their lifetime ^1^. In the 1930s, a study from Norway of nurses working in a TB hospital, a high-risk population, showed that nurses with a positive TST prior to their employment at the hospital had a 96% risk reduction of developing active TB compared to nurses who had a negative TST ^134^. A review of 18 prospective clinical trials from 2012 confirmed this with a 79% reduced risk of progression to active TB after reinfection in individuals with LTBI compared to previously non-infected people ^135^. However, LTBI in itself poses a risk to the development of active TB when control of *M. tuberculosis* is lost, for example because of age or immunosuppression. Moreover, it has been described that the rate of reinfection TB, defined as a recurrent TB episode with a different strain, in individuals who had previously already developed active TB disease and had successfully been treated, is higher than the rate of new TB cases in the population, suggesting that people who had TB once are at a strongly increased risk of developing TB when reinfected ^136^. Indeed, this underlines the variability and importance of individual human immune systems to cope with the infection. One interesting attempt to improve vaccine research is thus to identify correlates of protection by studying the protective immune response in people with LTBI ^51^. Simultaneously, new vaccine candidates are often designed following two simple principles to either make BCG more immunogenic by introducing strong immunogens such as ESAT-6 or render *M. tuberculosis* attenuated by the deletion of important virulence factors ^4^. Given the major advances that have been made recently in the genomic and cellular characterization of virulence factors and virulence strategies of the members of the MTBC and closely related tubercle bacilli, such as *M. canettii*, the challenge is now to transpose this knowledge into practice concerning vaccine research. There are some promising leads, which include a number of recombinant BCG strains that show improved protection in preclinical models ^86, 113, 137, 138^. In addition, some recombinant BCG strains have been or are presently in clinical trials ^72, 139^, and very recent results have also suggested that revaccination with BCG has a positive impact against infection with *M. tuberculosis* ^140^. Alternatively, there is hope that rationally attenuated *M. tuberculosis* strains may induce better protection than standard BCG vaccination, as suggested by different preclinical studies ^46, 141^. The fact that the attenuated *M. tuberculosis* strain MTBVAC is in clinical evaluation is an encouraging sign that attenuated *M. tuberculosis* strains may be part of the future vaccine strategies against *M. tuberculosis* infection and TB disease.

From a more upstream research prospective, the identification of novel virulence factors of pathogenic mycobacteria will certainly continue and might provide new perspectives for vaccine research. Several mycobacterial species that have been considered as most closely related to *M. tuberculosis*, such as *M. marinum* or *M. kansasii*, have been used to identify novel mycobacterial virulence factors ^142– 145^. However, according to the most recent genome analyses, several new, recently described mycobacterial species are much more closely related to *M. tuberculosis* than *M. marinum* and *M. kansasii* ^146, 147^. Indeed, several virulence factors, such as the fumarate reductase locus, the SL locus, or numerous toxin–antitoxin systems that have been considered as being present only in *M. tuberculosis* and/or *M. canettii*, can also be found in this group of closely related mycobacteria, which constitute a common clade with MTB that was named MTB-associated phylotype (MTBAP) ^147^. Future research will show if some of these virulence factors might have importance in vaccine research.
